# An inventory of Canadian pregnancy and birth cohort studies: research in progress

**DOI:** 10.1186/1471-2393-12-117

**Published:** 2012-10-29

**Authors:** Marie-Pier Joly, Michel Boivin, Anne Junker, Alan Bocking, Michael S Kramer, Stephanie A Atkinson

**Affiliations:** 1 Department of Sociology, University of Toronto, Toronto, ON, Canada; 2 École de psychologie, Université Laval, Quebec, PQ, Canada; 3 Department of Pediatrics, University of British Columbia, Vancouver, BC, Canada; 4 Department of Obstetrics & Gynecology, University of Toronto, Toronto, ON, Canada; 5 Departments of Pediatrics and of Epidemiology, Biostatistics and Occupational Health, McGill University, Montreal, PQ, Canada; 6 Department of Pediatrics, McMaster University, Hamilton, ON, Canada

**Keywords:** Birth and pregnancy cohort, Maternal health, Infant growth, Child mental development, Inventory

## Abstract

**Background:**

A web-based inventory was developed as a voluntary registry of Canadian pregnancy and birth cohort studies, with the objective to foster collaboration and sharing of research tools among cohort study groups as a means to enrich research in maternal and child health across Canada.

**Description:**

Information on existing birth cohort studies conducted in Canada exclusively or as part of broader international initiatives was accessed by searching the literature in PubMed and PsychInfo databases. Additional studies were identified by enquiring about the research activities of researchers at Canadian universities or working in affiliated hospitals or research centres or institutes. Of the fifty-eight birth cohort studies initially identified, forty-six were incorporated into the inventory if they were of a retrospective and/or prospective longitudinal design and with a minimum of two phases of data collection, with the first period having occurred before, during, or shortly after pregnancy and had an initial study sample size of a minimum of 200 participants.

Information collected from each study was organized into four main categories: basic information, data source and period of collection, exposures, and outcome measures and was coded and entered into an Excel spreadsheet. The information incorporated into the Excel spreadsheet was double checked, completed when necessary, and verified for completeness and accuracy by contacting the principal investigator or research coordinator. All data collected were then uploaded onto the website of the Institute of Human Development Child and Youth Health of the Canadian Institutes of Health Research. Subsequently, the database was updated and developed as an online searchable inventory on the website of the Maternal, Infant, Child and Youth Research Network.

**Conclusions:**

This inventory is unique, as it represents detailed information assembled for the first time on a large number of Canadian birth cohort studies. Such information provides a valuable resource for investigators in the planning stages of cohort studies and identifying current research gaps.

## Background

Prospective longitudinal pregnancy or birth cohort studies exist in many countries to investigate the effect of prenatal, pregnancy, and early postnatal exposures and interventions on maternal health, pregnancy outcomes, and long-term child health, social adjustment, and adult chronic disease. Many such studies are designed to investigate the linkages between environmental and genetic factors and health and disease outcomes in both mother and child. The emergence of several birth cohort studies in Canada prompted two meetings in 2009 that fostered interaction among researchers conducting such studies. The Workshop of the Canadian Birth Cohort Research Network sponsored by the Maternal, Infant, Child and Youth Research Network (MICYRN) had the objective to develop linkages between the existing Canadian birth cohort studies to facilitate interactions and improve the potential for research collaborations [1]. The Workshop on Canadian Children’s Environment and Health Research sponsored by Institute of Human Development, Child and Youth Health (IHDCYH) of the Canadian Institutes of Health Research (CIHR) and Health Canada was held to review the (then) current status and future needs for pregnancy/birth cohort studies with measures of exposure to agents of the physicochemical environment.

Subsequent to these initial meetings, plans were formulated through a partnership between MICYRN, CIHR-IHDCYH and the Strategic Knowledge Cluster on Early Childhood Development (SKC-ECD) to plan and further develop the inventory of seventeen identified birth cohort studies that participated in the MICYRN workshop. Such birth cohort inventories exist in Europe [2]. The initial inventory was then updated [3] and recently expanded to the web-based inventory described in this paper. The actual inventory represents a voluntary registry of Canadian birth cohort studies with the express purpose to serve the research community in its future applications in support of enriching research in maternal and family health as well as broader contexts of well-being such as social capital outcomes.

## Construction and content

### Study selection

The search strategy was not that of a systematic review. Two search strategies were used to identify birth cohort studies conducted in Canada exclusively or as part of broader international initiatives. The first strategy was a keyword search of the existing literature in the databases of PubMed and PsychInfo using various combinations of the following terms “Canada,” “birth cohort,” “follow up,” “longitudinal studies,” “birth,” “pregnancy,” “infant,” “newborn,” “child,” “maternal,” “prenatal,” “postnatal,” “health,” and “development.” Articles that provided detailed information on a birth cohort study and met the established inclusion criteria were carefully read. The second search strategy involved an enquiry about the research activities of research centers, institutes affiliated with Canadian universities or hospitals and researchers at Canadian universities, primarily in departments of obstetrics and gynecology, pediatrics, psychology, and epidemiology/community health.

Studies were included in the inventory if they were of a retrospective and/or prospective longitudinal design, with a minimum of two phases of data collection. Cross-sectional studies with retrospective data related to pregnancy and birth were not included. The first period of data collection had to have occurred before, during, or shortly after pregnancy including data retrospectively collected. Studies that had early childhood (beyond birth) as the first period of data collection were excluded. However, longitudinal studies conducted among children were included when retrospective data on pregnancy or young infant were collected. The initial subject sample size had to be a minimum of 200 participants. Smaller sample sizes were only included for studies on populations experiencing specific medical conditions, such as in premature babies and on women experiencing depression during pregnancy. For the purpose of this project, no limitation was placed on the number of years participants were enrolled in the study.

### Information collected

Information collected from each study was organized into four main categories: basic information, data source and period of collection, exposures, and outcome measures. Basic information included cohort study title, the principal investigator(s), lead institute(s), enrolment year, enrolment status of the study, study design, initial sample size of mothers, fathers and children, inclusion and exclusion criteria for participants, gestational age at enrolment, source population, coverage, expected duration of follow-up, sample size at each follow-up, and cohort website address.

Data sources included administrative databases, questionnaires, and bio-genetic samples. Data collection periods were categorized into first, second, or third trimester of pregnancy, at birth, 0 to 6 months, 7–18 months, 19–60 months, or 5 or more years after birth. The bio-genetic samples collected included blood, urine, hair, and saliva from mothers, fathers, infants, and children. Samples of placenta, meconium, breast milk, umbilical cord and umbilical cord blood were also noted. Blood samples were indicated as serum, plasma, whole blood, red cells, or white cells.

Exposures were subdivided into social environment (demographic characteristics of parents, family structure and composition, socio-economic status, neighbourhood characteristics, social support, and child care), school environment, social policy, natural environment, indoor environment, and food quality.

Outcomes were subdivided into birth, child, and maternal outcomes. For each of these categories, information was divided into the following subcategories: preterm birth; fetal growth; birth defects; severe neonatal morbidity; neonatal mortality; child growth; acute and chronic illness among children; neurocognitive development; children behavioural problems and mental illness; language development; pregnancy complications; mode of delivery; severe maternal morbidity; and maternal mental health.

Information obtained from each study was coded when necessary and entered into an Excel spreadsheet. Specific variables were assigned to the status of a study (“ongoing,” “in development” or “completed”) and source population (“selected” (e.g., populations at high-risk or representing a specific medical condition)), “hospital-based,” “region-based,” “nation-based,” or “multi-national”). Whether the sample was (a) of convenience, (b) population-based but not necessarily representative, or (c) representative (corrected for biases using population weights) was not categorized. “Y” or “N,” designating “yes” or “no,” was attributed to the types and periods of data source collection, exposures, and outcomes studied. The validity and reliability of these studies have nonetheless not been evaluated.

The information incorporated into the inventory was verified and validated by contacting the principal investigator or research coordinator by phone or email. A period of one month was allocated to the validation of information, after which a second email was sent to researchers who had not yet responded. In the fall of 2009, all data collected were uploaded on the website of IHDCYH [3].

### Web application of the inventory

Data contained in the Excel spreadsheet were modified according to program established for on-line access. Data in the inventory were updated in August 2010 through contact with the principal investigators or research coordinators of the studies logged in the system.

### Results

**Figure 1:**
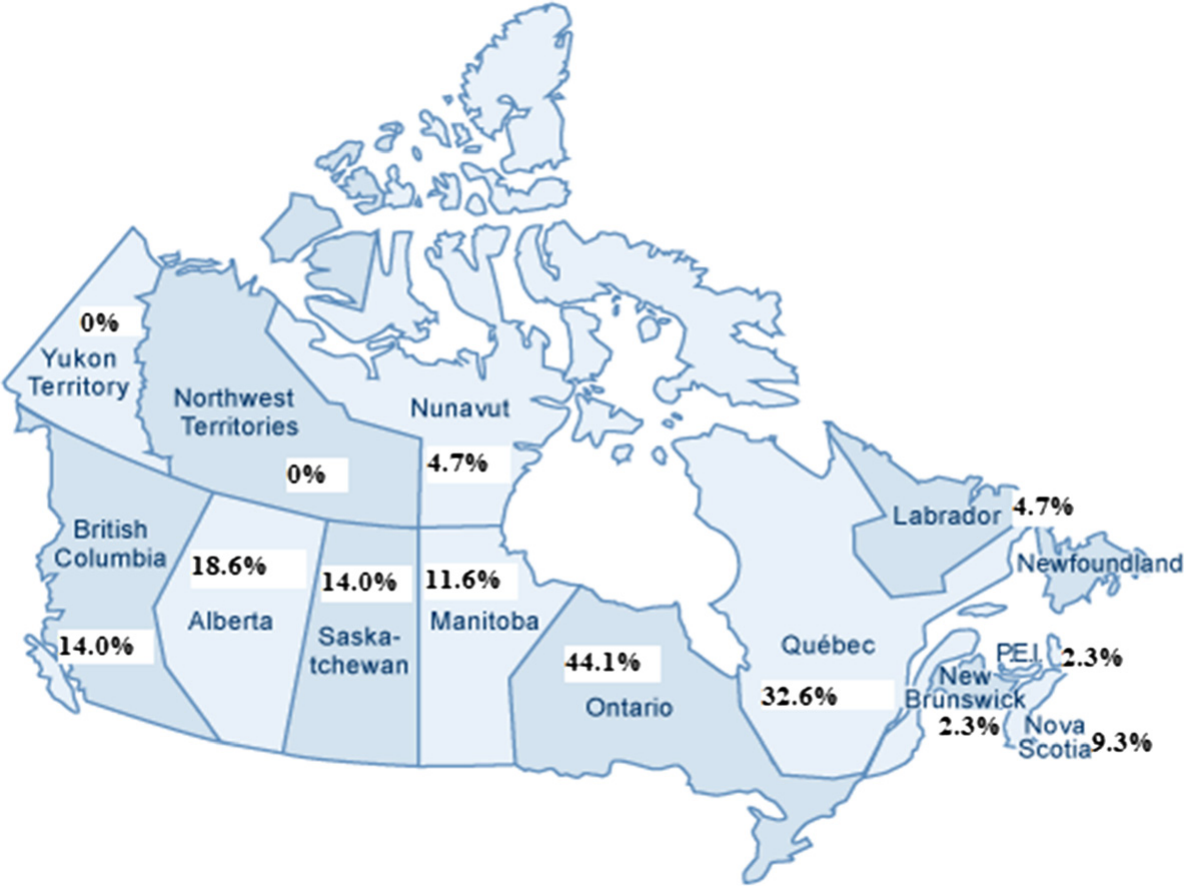
**Percent of birth cohort studies conducted by province and territory.** Some studies cover more than one province or territory.

*Profiles of cohort studies:* Of the 58 pregnancy and birth cohort studies initially identified, 46 were validated and incorporated into the inventory. The majority of these studies are either ongoing or in development; only 11 had been completed at the time the inventory was created. In total, these 46 birth cohort studies involve over 950,000 mothers, 24,000 fathers, and 1 million children; 78% of the studies have an initial sample size greater than 1000 participants. To our knowledge, only two studies were conducted among participants from a previous cohort, both of which are included in the inventory. The Emigarde project [4] drew on subjects from the Montreal Prematurity Project [5,6] and the SAGE nested case control study drew on the SAGE study [7]. The most common source population is region-based (61%), followed by hospital-based (16%), selected (9%), nation-based (7%) and multi-national (7%). Coverage of geographical locations within Canada was noticeably diverse. As demonstrated in Figure 1, most studies have collected data in the provinces of Ontario (44.1%) and Quebec (32.6%). The percentage of studies conducted in Saskatchewan, Manitoba, Alberta, and British Columbia range from 11.6-18.6%. Considerably less attention is devoted to the Eastern provinces and the Territories. The majority of participants have been enrolled over the last two decades, with the exception of two studies for which enrolment started in late 1970s.

**Figure 2:**
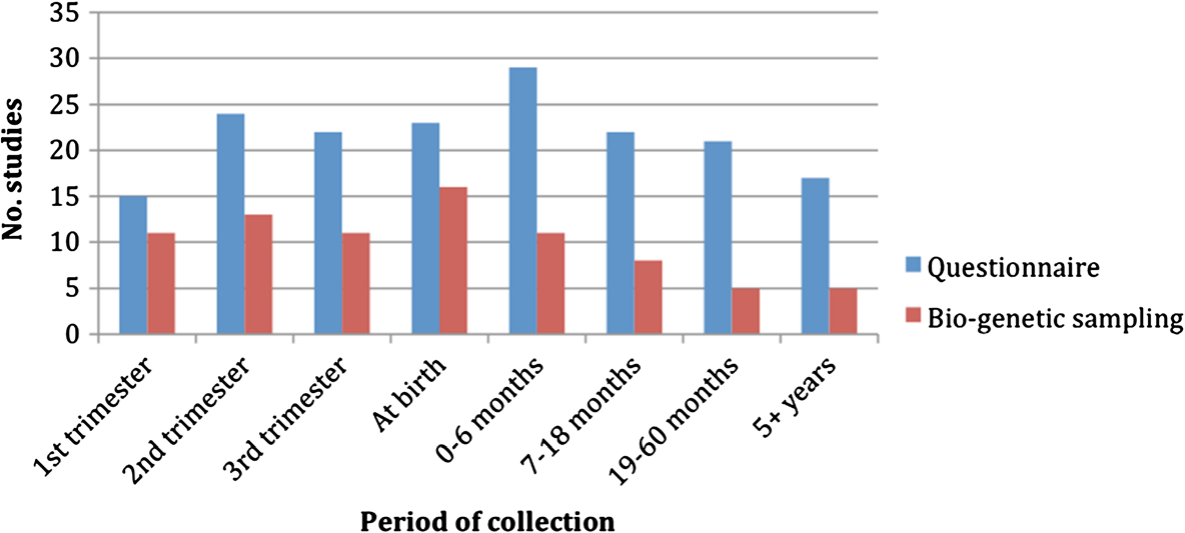
**Periods of data collection used for questionnaires and bio-genetic sampling in birth cohort studies.** Included studies were required to use at least two periods of data collection.

*Data collection*: Although certain studies had only one means to collect their data, several studies used more than one method. While 37% of birth cohort studies rely on administrative databases such as the *Quebec Pregnancy Registry* and the *London Perinatal Database Retrospective*, 87% of studies collect their data through questionnaires and 54% of studies include bio-genetic samples. The most common period for data collection with questionnaires was 0–6 months after birth (63%), and the most common period for collecting bio-genetic samples was at birth (34.8%) (Figure 2).

**Figure 3:**
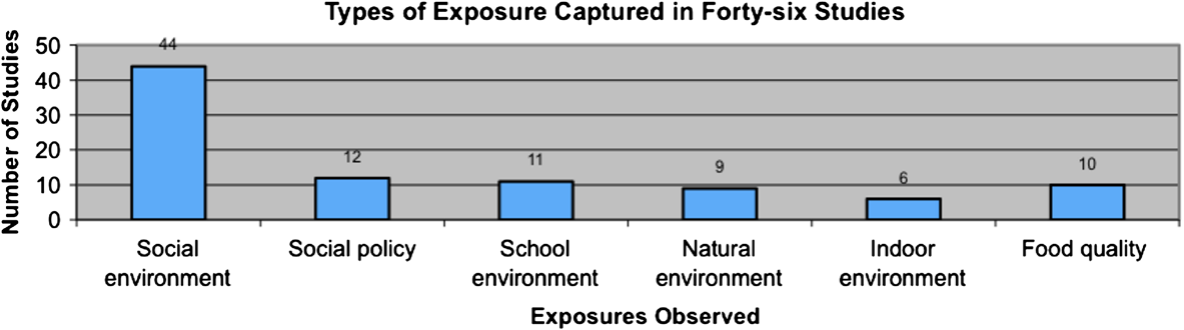
Categories of environmental exposure settings in Canadian birth cohort studies.

The environmental exposures varied widely across studies (Figure 3). Forty-four studies (95%) included measures of social environment, while natural and indoor environment measures were included only in 9 and 6 studies, respectively.

Key outcome measures included pregnancy complications (43 studies), severe maternal morbidity (30 studies), birth defects (31 studies), and neonatal morbidity/mortality (34 studies), neurocognitive development (26 studies), and behavioural problems and mental illness among children (27 studies).

The types of bio-genetic samples collected at various intervals in pregnancy and infancy varied across studies (Table 1). Umbilical cord blood was the bio-genetic sample collected by most studies (30.4%), while maternal blood was collected over various time periods. Bio-genetic samples have seldom been collected from fathers.

**Table 1:** Number of birth cohort studies that collected bio-genetic samples at various time periods

**Biogenetic sample**	**Time period; no. (%) of studies**							
	**n=46**							
	**1^st^**	**2^nd^**	**3^rd^**	**At**	**0-6**	**7-18**	**19-60**	**5+**
	**Trimester**	**Trimester**	**Trimester**	**Birth**	**Months**	**Months**	**Months**	**Years**
**Maternal blood**	8	11	9	7	3	4	2	1
	(17.4%)	(23.9%)	(19.6%)	(15.2%)	(6.5%)	(8.7%)	(4.3%)	(2.2%)
**Paternal blood**	1	1	1	-	-	-	1	3
	(2.2%)	(2.2%)	(2.2%)				(2.2%)	(6.5%)
**Cord blood**	-	-	-	14	-	-	-	-
				(30.4%)				
**Placenta**	-	-	-	9	-	-	-	-
	-	-	-	(19.6%)	-	-	-	-
**Umbilical cord**	-	-	-	3	-	-	-	-
-		-	-	(6.5%)	-	-	-	-
**Offspring blood**	-	-	-	-	4	2	3	4
					(8.7%)	(4.3%)	(6.5%)	(8.7%)
**Meconium**	-	-	-	5	-	-	-	-
				(10.9%)				
**Breast milk**	-	-	-	-	6	**-**	**-**	-
					(13.0%)			
**Maternal urine**	5	5	5	2	-	-	-	-
	(10.9%)	(10.9%)	(10.9%)	(4.3%)				
**Paternal urine**	1	-	-	-	-	-	-	-
	(2.2%)							
**Offspring urine**	-	-	-	**-**	2	1	1	2
					(4.3%)	(2.2%)	(2.2%)	(4.3%)
**Maternal hair**	3	2	1	3	2	1	1	-
	(6.5%)	(4.3%)	(2.2%)	(6.5%)	(4.3%)	(2.2%)	(2.2%)	
**Paternal hair**	-	-	-	-	-	1	-	-
-		-	-	-	-	(2.2%)	-	-
**Offspring hair**	-	-	-	1	1	1	1	1
				(2.2%)	(2.2%)	(2.2%)	(2.2%)	(2.2%)
**Maternal saliva**	2	1	1	-	2	3	1	1
	(4.3%)	(2.2%)	(2.2%)		(4.3%)	(6.5%)	(2.2%)	(2.2%)
**Paternal saliva**	-	1	-	-	-	-	1	1
		(2.2%)					(2.2%)	(2.2%)
**Offspring saliva**	-	-	-	**-**	1	3	3	2
					(2.2%)	(6.5%)	(6.5%)	(4.3%)
**Maternal nails**	-	-	1	-	-	-	-	-
			(2.2%)					
**Paternal nails**	-	-	-	-	-	-	-	-
**Offspring nails**	-	-	-	**-**	-	-	-	-

## Utility

The web application online searchable inventory not only facilitates the search for information, but also the management of the inventory. Studies can be retrieved by selecting a particular type of biological sample, exposure, or outcome of interest. The search can be restricted by choosing a combination of measures under these three categories or by entering a keyword*.* The inventory also represents an important tool to foster potential and eventual collaborations among researchers. Among the array of information available from this inventory is information about the possibility for sharing the biobank and for research collaboration. Interested researchers can explore the complete inventory to examine such possibilities.

## Discussion

The current online cohort inventory provides key information stemming from 46 pregnancy and birth cohort studies that have incorporated a large number of subjects studied across diverse geographical locations in Canada. Types of exposures, outcomes, and biological samples are the key information elements available in the inventory. While nearly one million mothers and their offspring have been recruited in total across the 46 studies, not all subjects would be available for future contact. However, for studies still ongoing, re-consent might be possible if additional follow-up was desirable.

This inventory is unique, as it represents detailed information assembled for the first time on a large range of Canadian birth cohort studies. As well, the content and format is aligned with inventories from Europe [2] and others designed with a genetics focus including the Public Population Project in Genomics (P^3^G), which supports the development and harmonization of epidemiological projects in genomics [8]. This alignment should facilitate assembly of information across existing published cohort study inventories.

The inventory also reveals major gaps in research. While most studies have collected data on the social environment of participants, data on the natural and indoor environment has been infrequently assessed. Despite the geographical coverage of studies extending across Canada, most are from Ontario or Quebec, and few include populations from the Eastern provinces or the Territories. In addition, few studies had immediate plans for genetic analysis, although some indicated banking of biological samples for possible future analysis.

Output from the cohort workshops clearly identified the sharing of research tools as a key element that might be accommodated in future development of the online inventory. To evaluate that potential, we conducted a literature search of selected studies represented in the current inventory to determine the extent to which measurement tools currently employed were consistent across studies. This was accomplished by reviewing papers published by the cohorts that cited the measurement tools used. Researchers have drawn upon an array of scales, tests, and assessments to measure health outcomes (Table 2). The child and maternal outcome measures studied shared several published questionnaires and scales (Table 2).

**Table 2:** Outcomes measures from selected studies

**Name of study**	**Birth outcomes measures**	**Child outcomes measures**	**Maternal outcomes measures**
Better Beginnings, Better Futures [9]		Social Skills Rating Scales [10]; Revised Ontario Child Health Study [11]; Peabody Picture Vocabulary Test [12]; Wechsler Intelligence Scale for Children- Revised [13]; Wide Range of Achievement Test [14]; Scale of Reading Attitude [15]	
Community Perinatal Care Study [16]		Pediatric Evaluation of Developmental Status (PEDS) [17]; Child Social Competence Scale [18]; SF-8 Health Survey [19]	Edinburgh Posnatal Depression Scale (EPDS) [20]
Ice Storm Project [21-23]		Mental Scale of the Bayley Scales of Infant Development (2nd Ed.) [24]; MacArthur Communicative Development Inventory (MCDI) [25]; Wechsler Preschool and Primary Scale of Intelligence-Revised (WPPSI-R) [26]; Peabody Picture Vocabulary Test-Revised (PPVT-R) [27]	Impact of Event Scale-Revised (IES-R) [28]; General Health Questionnaire (GHQ) [29]; Edinburgh Postnatal Depression Scale (EPDS) [20]
The International Randomized Term Breech Trial [30,31]		Ages and Stages Questionnaire (ASQ) [32]	Edinburgh Posnatal Depression Scale (EPDS) [20]
Ottawa Prenatal Prospective Study (OPPS) [33-35]	Brazelton Neonatal Assessment Scale [36]	Bayley Scales of Infant Development [37]; Reynell Developmental Language Scales [38]; Prechtl assessment [39]	
The Ontario Mother and Infant Study (TOMIS) III [40]			Edinburgh Posnatal Depression Scale (EPDS) [41]
Victimization: A Newly Recognized Outcome of Prematurity [42]		McCarthy Scales of Children’s Abilities [43]Hunttenlocher neurological task test [44]	
Family Atherosclerosis Monitoring in Early Life (FAMILY) [45]		Habitual Activity Estimation Scale (HAES) [46]	Food Frequency Questionnaire [47]

The selected review of measurement tools employed in the cohort studies included in the inventory revealed some consistency for measures of infant and child neurocognitive, education and social skills and of maternal depression. Clearly, there is great opportunity for sharing of other validated tools, such as quantitative assessment of dietary intake, physical activity, or relevant environmental influences.

## Conclusions

The current inventory represents only a first step. MICYRN and its partners, including the SKC-ECD will update and expand the inventory. Future expansion could include a repository for measurement tools, such as questionnaires and analytical methods, which could be shared with other studies and available upon registration in the MICYRN Birth Cohort Inventory. Such collaborations could improve validity and quality assurance of measurement tools. Finally, linkage of birth cohort databases to health care and other databases could also be facilitated by MICYRN to provide expanded opportunities for in-depth analysis.

## Availability and requirements

The online inventory is publically available and can be accessed at http://www.micyrn.ca/Networks.asp#2. The inventory can be searched at http://www.micyrn.ca/databases/cohortsearch.asp by type of Biological Samples, Exposures or Outcomes, and by Keyword. There is a link to a spreadsheet view of the complete inventory and a more detailed view of all data being collected in the complete inventory. Researchers are welcome to contribute to the development of the inventory by registering their cohort study, which meet our criteria of inclusion, at the following link http://www.micyrn.ca/databases/asplogin/CohortLogin.asp#2. Submitted forms return to the MICYRN Secretariat and are reviewed prior to entry into the Inventory to ensure they meet the original criteria set for study selection as described in the Background. Future expansion of the inventory will include an international scope.

## Abbreviations

MICYRN: Maternal, Infant, Child and Youth Research Network; IHDCYH: Institute of Human Development, Child and Youth Health; CIHR: Canadian Institutes of Health Research; SKC-ECD: Strategic Knowledge Cluster on Early Childhood Development; P^3^G: Public Population Project in Genomics.

## Competing interests

The authors declare that they have no competing interests.

## Authors’ contributions

SA initiated the pregnancy and Canadian birth cohort network and inventory and was responsible for the development of the manuscript and oversight of its completion and submission. AB was co-initiator of cohort network and reviewed the manuscript. MB contributed to the development of the inventory and reviewed the manuscript. M-P J conducted the literature search to identify the expanded cohort database and contributed to the establishment of the inventory within the Canadian Institutes for Health Research (CIHR) Institute of Human Development, Child and Youth Health (IHDCYH) and took a primary role in the writing and revisions of the manuscript. AJ facilitated the establishment of the online searchable inventory on the website of the Maternal, Infant, Child and Youth Research Network and reviewed the manuscript. MK facilitated the development of the inventory as a project within the CIHR IHDCYH. All authors read and approved the final manuscript.

## Authors’ information

M-P J contributed initially to this project when a study analyst for the Institute of Human Development, Child and Youth Health at the Canadian Institutes of Child Health in Ottawa, Canada. She is currently a PhD student in the department of sociology at the University of Toronto.

## Pre-publication history

The pre-publication history for this paper can be accessed here:

http://www.biomedcentral.com/1471-2393/12/117/prepub
